# Gallbladder Volvulus Mimicking an Intra-abdominal Malignancy in an Elderly Patient on Warfarin: A Case Report

**DOI:** 10.7759/cureus.85771

**Published:** 2025-06-11

**Authors:** Belinda Edokpolo, John W Shaw

**Affiliations:** 1 Hospital Medicine, Hutchinson Regional Medical Center, Hutchinson, USA; 2 General and Colorectal Surgery, Hutchinson Regional Medical Center, Hutchinson, USA

**Keywords:** anticoagulation therapy, biliary torsion, complicated acute cholecystitis, ct (computed tomography) imaging, elderly patient, epigastric mass, gallbladder volvulus, hemorrhagic cholecystitis, ultrasound findings, warfarin

## Abstract

Gallbladder volvulus is a rare but potentially fatal condition that may present with symptoms mimicking intra-abdominal malignancy, including constitutional symptoms and mass-like imaging findings. We present the case of an 85-year-old female on chronic anticoagulation with warfarin, who was evaluated for an epigastric mass initially suspected to be lymphoma or colon cancer. CT imaging revealed a large, heterogeneous mass in the epigastric region, while ultrasound demonstrated significant gallbladder distention, wall thickening, and pericholecystic fluid. This report highlights the challenges in obtaining an accurate diagnosis and stresses the urgent need for swift imaging and intervention to distinguish between surgical emergencies and oncologic-related conditions in elderly patients.

## Introduction

Gallbladder volvulus is a rare but potentially fatal surgical condition that involves torsion of the gallbladder along its mesentery, leading to vascular compromise, necrosis, and eventual perforation if not promptly treated. First described by Wendel in 1898 [1], fewer than 600 cases have been reported in the literature to date [2]. The condition predominantly affects elderly women and is often associated with anatomical predispositions, such as a long mesentery, loss of visceral fat, and hepatic atrophy, which increase gallbladder mobility [2-4]. Clinical symptoms frequently resemble acute cholecystitis, bowel obstruction, or intra-abdominal malignancy, which complicates preoperative diagnosis [4,5]. In patients receiving anticoagulation therapy, including warfarin, the typical inflammatory response may be blunted, further obscuring the clinical picture and delaying recognition [6,7]. The nonspecific nature of symptoms and overlapping imaging findings necessitates a high index of suspicion to enable timely surgical intervention.

## Case presentation

An 85-year-old female with a history of paroxysmal atrial fibrillation on chronic warfarin, hypertension, and celiac disease presents as a transfer from an outlying facility for evaluation of an epigastric mass. She reported one week of right-sided abdominal pain, nausea, night sweats, anorexia, fatigue, and constipation. She denied weight loss or gastrointestinal bleeding. Vital signs on admission were a heart rate of 110 beats per minute, a respiratory rate of 19 breaths per minute, a blood pressure of 90/60 mmHg, and an oxygen saturation of 97%. The physical exam indicated tenderness in the right upper quadrant, with no signs of palpable lymphadenopathy or jaundice. Laboratory findings included leukocytes: 20,600; sodium: 119 mEq/L; and INR: 6.6 (therapeutic goal: 2.0-3.0).

**Figure 1:**
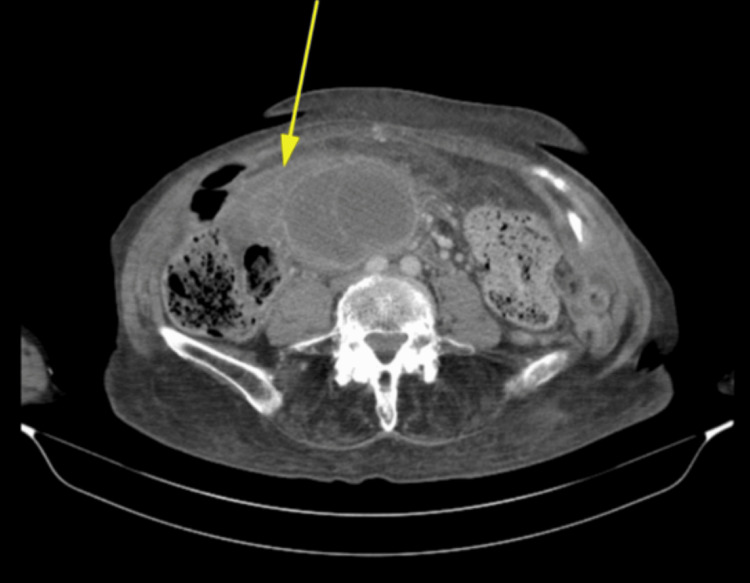
Axial CT of the Abdomen Showing a Large Epigastric Mass Displacing Adjacent Bowel Loops Axial CT image of the abdomen and pelvis demonstrating a large, well-defined, heterogeneous mass in the epigastric region, extending toward the right upper quadrant (arrow). The mass displaces adjacent bowel loops and was initially suspicious for intra-abdominal malignancy.

**Figure 2:**
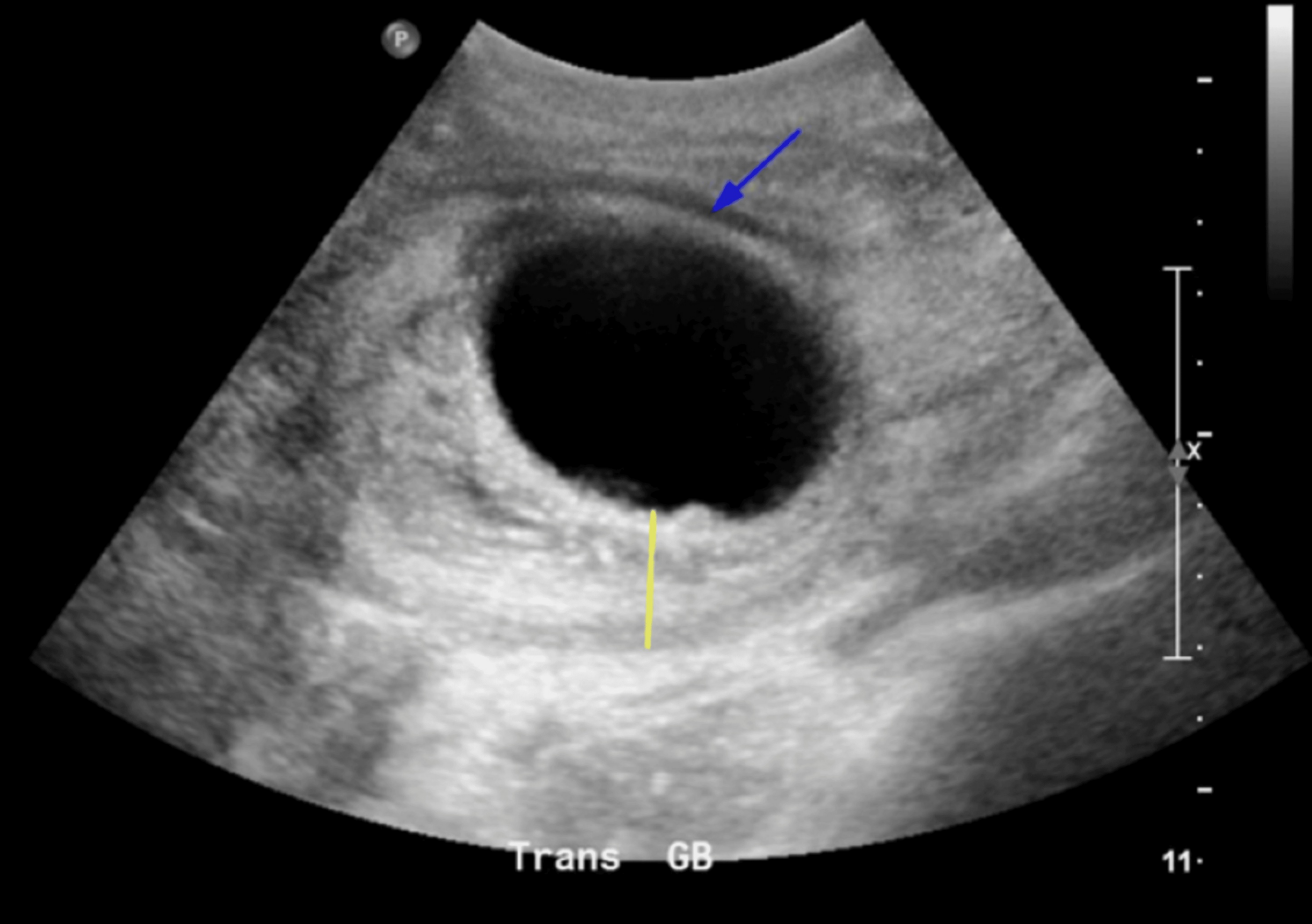
Transverse Ultrasound of the Gallbladder Demonstrating Wall Thickening and Pericholecystic Fluid Transverse ultrasound of the gallbladder showing marked distention, previously measured at 11 cm (not to scale in this image). Wall thickening is demonstrated (yellow line, not to scale), and pericholecystic fluid is noted (blue arrow), consistent with hemorrhagic cholecystitis.

A CT abdomen/pelvis revealed a large, well-defined mass in the epigastric region, extending toward the right upper quadrant and displacing adjacent bowel structures, which contributed to the patient's abdominal distension and clinical presentation (Figure 1). The gallbladder ultrasound revealed a markedly distended gallbladder (approx. 11 cm in length) with wall thickening (up to 3.3 cm). Findings also included hyperechoic shadowing areas suggestive of gallstones, pericholecystic fluid, and echogenic debris (Figure 2). Based on these findings, acute cholecystitis was suspected.

The patient received anticoagulation reversal with intravenous vitamin K and fresh frozen plasma. Due to clinical deterioration, she underwent an emergent open cholecystectomy, which revealed gallbladder torsion and hemorrhagic cholecystitis with necrosis. Surgeons found no gross or histological evidence of malignancy on pathology. Intraoperative images were not obtained due to the urgent nature of the surgery, which necessitated prioritizing patient stabilization. The patient recovered without complications and was discharged in stable condition on postoperative day 5, with plans for outpatient follow-up.

## Discussion

Gallbladder volvulus is a rare but critical surgical emergency, with fewer than 600 cases reported globally [2]. It most commonly affects elderly women, particularly those with predisposing anatomic features such as a long mesentery, hepatic atrophy, or decreased visceral fat, all of which enhance gallbladder mobility and increase the risk of torsion [2,3]. When torsion occurs around the cystic duct and artery, it compromises vascular supply and can rapidly progress to ischemia, hemorrhage, necrosis, and, if left untreated, perforation [4].

In this case, the patient's presentation with constitutional symptoms and a large epigastric mass on CT raised early concern for intra-abdominal malignancy. However, a targeted ultrasound revealed hallmark inflammatory findings: a markedly distended gallbladder, wall thickening, echogenic intraluminal debris, gallstones, and pericholecystic fluid. Notably, the ultrasound lacked features commonly associated with gallbladder carcinoma, such as intraluminal polypoid masses, irregular wall thickening, or evidence of hepatic invasion [5,6]. The absence of these malignancy-associated features helped redirect diagnostic considerations toward an inflammatory etiology.

Despite advances in cross-sectional imaging, preoperative diagnosis of gallbladder volvulus remains challenging. Radiologic clues, such as the "floating gallbladder" sign, horizontal orientation of the organ, or the cystic duct knot sign, can suggest torsion but are often subtle and easily overlooked [8,9]. Magnetic resonance cholangiopancreatography (MRCP) may support diagnosis in select cases [8], though its use is limited in acute care settings. As with most reported cases, a definitive diagnosis in our patient was made intraoperatively [10,11].

A complicating factor was the patient's markedly elevated INR of 6.6 due to chronic warfarin use. Anticoagulation can blunt the peritoneal signs of inflammation and simultaneously elevate the risk of hemorrhagic cholecystitis, both of which can obscure the diagnosis and mimic malignancy on imaging [6,12]. Prior reports describe how coagulopathy contributed to diagnostic uncertainty by masking classical inflammatory findings [6,13]. The surgical team observed hemorrhagic necrosis of the gallbladder during the procedure, but they did not obtain intraoperative photos due to the emergent nature of the operation. Pathology confirmed the absence of malignancy, validating the preoperative suspicion of a non-neoplastic etiology.

Multiple authors have proposed classifications based on the degree of torsion, with implications for presentation and severity [14]. Moreover, some cases of gallbladder volvulus are discovered incidentally during surgery for presumed acute cholecystitis, emphasizing the importance of intraoperative vigilance when preoperative findings are inconclusive [15,16].

While laparoscopic cholecystectomy is generally the preferred approach for stable patients due to its favorable recovery profile [17,18], specific clinical contexts necessitate an open approach. In our case, the patient's hemodynamic instability, anticoagulation status, and friable tissue architecture prompted the surgical team to proceed with open cholecystectomy. This approach enabled safer dissection and better control of intraoperative bleeding, particularly in anatomically distorted or inflamed surgical fields [19,20].

This case reinforces key diagnostic and management principles. Clinicians should maintain a high index of suspicion for gallbladder volvulus in elderly or anticoagulated patients presenting with abdominal pain and inconclusive imaging. Prompt surgical intervention remains the cornerstone of treatment and can significantly reduce the risk of morbidity and mortality in this vulnerable population.

## Conclusions

Clinicians should consider gallbladder volvulus in elderly patients presenting with abdominal pain and inconclusive imaging results, especially when anticoagulation therapy masks typical signs of inflammation. This case highlights the importance of maintaining clinical vigilance and acting quickly with surgical intervention when indicated. Recognizing this rare condition more readily can help reduce diagnostic delays and improve patient outcomes.
